# Symptom-based clusters in people with post-COVID-19 condition (PCC)

**DOI:** 10.1186/s12967-026-08582-4

**Published:** 2026-07-11

**Authors:** Charlotte Kuczyk, Christoph Herrmann-Lingen, Maike Stolz, Christian Krauth, Birte Burger, Jona Theodor Stahmeyer, Mariel Nöhre, Aline Debener, Christopher Käufer, Franziska Richter, Carlotta Derad, Martin Hellmich, Martina de Zwaan

**Affiliations:** 1 Department of Psychosomatic Medicine and Psychotherapy, Hannover Medical School, Hannover, Germany; 2 Department of Psychosomatic Medicine and Psychotherapy, University Medical Center Göttingen, Göttingen, Germany; 3 Institute for Epidemiology, Social Medicine and Health Systems Research, Hannover Medical School, Hannover, Germany; 4 Center for Health Economics Research (CHERH), Hannover, Germany; 5 Health Services Research Unit, AOK Niedersachsen, Hannover, Germany; 6 Department of Pharmacology, Toxicology and Pharmacy, University of Veterinary Medicine, Hannover, Germany; 7 Department of Medical Statistics, University Medical Center Göttingen, Göttingen, Germany; 8 Center for Systems Neuroscience, Hannover, Germany; 9 Biomedical Research in Endstage and Obstructive Lung Disease Hannover (BREATH), German Center for Lung Research (DZL), Hannover, Germany

**Keywords:** COVID-19, Post COVID − 19 condition, Symptoms, Clusters

## Abstract

**Background:**

Identifying symptom clusters in post-COVID-19 condition (PCC) is a necessary step toward for developing more targeted therapeutic interventions for this heterogeneous condition. Therefore, the aim of this study was to identify symptom clusters based on 14 specific PCC symptoms, accounting for both symptom presence and impairment. The identified clusters were then compared with respect to sociodemographic, clinical, and psychological factors.

**Methods:**

A clinical sample of individuals with a PCC diagnosis lasting at least one year was included (final *n* = 1673). A two-step cluster analysis was performed to identify symptom clusters. Subsequent comparisons between clusters were performed using Mann-Whitney U tests for continuous variables and chi-square tests for categorical variables.

**Results:**

A total of four clusters were identified: two symptom burden clusters (*Systemic* (high burden) and *Few Symptoms* (low burden)) and two symptom-specific clusters (*Neurocognitive* and *Pain*). Participants in the *Systemic* (high burden) cluster exhibited the highest levels of psychological distress, reported the most severe fatigue, and were most frequently unemployed.

**Conclusion:**

In PCC, different symptom clusters can be identified that differ in terms of sociodemographic, clinical, and psychological factors. Future research using biomarker, imaging, and longitudinal designs is needed to determine whether these symptom-based clusters correspond to distinct biological subgroups.

**Supplementary Information:**

The online version contains supplementary material available at 10.1186/s12967-026-08582-4.

## Introduction

Although most patients recover within a few weeks after contracting COVID-19, some experience long-term consequences of COVID-19 infection, also known as post-COVID-19 condition (PCC). The definition of PCC encompasses a variety of symptoms and signs reported by patients after an initial SARS-CoV-2 infection [1]. PCC can cause a wide range of symptoms that can affect various organ systems, including the respiratory, cardiovascular, gastrointestinal, and nervous systems.

In this context, more than 200 different types of symptoms have been reported [2], with the most common symptoms including fatigue, post-exertional malaise (PEM), cognitive impairment, dyspnea, sleep disturbances, and muscle and joint pain [3–5]. The variety and heterogeneity of symptoms pose considerable challenges in treating PCC. Over the past few years, several studies have been conducted with the aim of better understanding the risk factors and pathophysiology of PCC in order to derive appropriate treatment strategies. To date, however, the pathophysiological mechanisms of PCC have not been clearly elucidated. So far, it has only been hypothesized that dysregulation of the immune system, inflammatory reactions, viral persistence, reactivation of pathogens in connection with the host microbiome, autoimmune processes, and activation of coagulation could contribute to the etiology of PCC [6, 7]. Accordingly, no specific pharmacological treatment or other intervention has yet been established for the treatment of PCC, and the treatment approach is exclusively symptomatic. Against the backdrop of an exclusively symptom-oriented treatment approach, there are already a few studies that have investigated the extent to which certain distinguishable symptom clusters/symptom phenotypes can be identified, taking into account the multitude of possible symptoms associated with PCC. A symptom cluster is defined as several symptoms that occur together, are associated with each other, and differ from other clusters. Some studies provide evidence of individual recurring symptom clusters [8, 9], although depending on the study, different sets of symptoms were examined over varying periods of time since infection and clustering was performed using different methods, which means that no uniform set of symptom clusters has yet been identified in the context of previous research.

Most previous studies, which also aimed to identify symptom clusters, mostly considered the presence or absence of various symptoms in their analyses, whereas the impairment caused by the respective symptoms was addressed to a lesser extent. However, recent advances in multi-omics research are beginning to bridge this gap by linking precise clinical impairments directly to underlying molecular signatures. At the transcriptomic level, alterations such as profound immune exhaustion and cellular senescence have been identified that clearly separate PCC patients from fully recovered individuals [10, 11]. Similarly, longitudinal proteomic profiling has demonstrated that specific plasma protein signatures can accurately predict the evolution of functional impairments, such as restrictive lung disease [12], and correlate strongly with objective biochemical severity scales [13].

Furthermore, precision phenotyping studies increasingly map distinct long-term clinical profiles - ranging from subjective symptom clusters like fatigue and sensory loss to objective pulmonary abnormalities - to specific early inflammatory predictors and local multi-omics signaturess [14, 15]. Despite these critical molecular insights, in daily clinical practice it remains essential to systematically assess both the subjective presence of symptoms and the actual degree of impairment they cause.

Therefore, the present study aims to investigate the extent to which specific symptom clusters can be identified in a large sample of patients who are still affected by PCC in the longer term, and consider a broad spectrum of symptoms. These clusters are formed not only on the basis of the presence of symptoms, but also on the degree of impairment caused by the respective symptoms. In addition, the extent to which the identified clusters are associated with sociodemographic, clinical, and psychological factors is analyzed.

The identification of possible symptom patterns or symptom phenotypes offers a way to divide the multitude of symptoms into structured patterns. This could create a framework for assessing symptom burden, setting treatment priorities, and developing targeted treatment approaches. Thus, there could be a shift from treating isolated symptoms to a more precise, targeted approach that focuses on the treatment of specific symptom phenotypes.

### Methods and materials

Individuals diagnosed with PCC were identified using health insurance data from the largest statutory health insurer in Lower Saxony, AOK Niedersachsen, which provides coverage for approximately 3 million people, accounting for almost 40% of the population in this region. Patient identification followed the procedures described in the VePoKaP (Care for post-COVID-19 condition in Germany from the perspectives of patients, informal caregivers and general practitioners) study protocol [16]. Individuals insured with AOK Niedersachsen who met the following inclusion criteria were invited by mail to participate in an online survey: (1) aged 18 years or older with a confirmed PCC diagnosis, as indicated by the ICD code U09.9! in their claims data in 2022 (outpatient billing records or a 2022 certificate of incapacity for work); 2) residence in Lower Saxony; and 3) continuous insurance coverage with AOK Niedersachsen since 2019. Patients under legal guardianship and AOK employees were excluded. A total of *N* = 26,438 individuals met the eligibility criteria. For budgetary reasons, *N* = 20,163 were randomly selected and contacted by mail. Of those invited, *N* = 2,159 (10.7%) provided informed consent and completed the survey. Of the 2,159 respondents, individuals who reported ongoing post-COVID-19 symptoms were included, resulting in a final sample of *N* = 1,673 participants for the subsequent statistical analyses. Ethical approval was obtained from the ethics committee of Hannover Medical School (reference number 11077_BO_K_2023). All participants provided their informed consent electronically regarding study participation and the use of their health insurance data. Furthermore, approval for the use of health insurance data according to § 75 SGB X (Social Security Code Book 10) was given by the competent supervisory authority.

## Measurement

### Symptom impairment

Patients were asked about the presence of 14 symptoms [17, 18] to determine whether each symptom was currently present. The symptoms assessed were fatigue, long recovery phase after light exertion (PEM), brain fog, difficulty concentrating, memory difficulties, chest pain, joint pain, muscle pain/cramps, headache, heart palpitations, shortness of breath, cough, sleep disorders, and loss of smell/taste. The responses were dichotomized, resulting in 14 binary variables indicating whether each symptom was currently present (1) or not (0).

For all symptoms reported as currently present, the degree of impairment was assessed using the following question: *“How severely do the following currently existing symptoms impair you?”* Responses were given on a five-point Likert scale (not at all, slightly, moderately, severely, very severely). For the subsequent main cluster analysis, 14 binary impairment variables were created by combining the impairment levels “not at all” and “slightly” into one category, and the impairment levels “moderately,” “severely,” and “very severely” into another.

### Other measurement instruments

#### Depression and anxiety

Depression and anxiety were assessed using the four-item Patient Health Questionnaire (PHQ-4) [19]. The PHQ-4 is an ultra-brief self-report screening instrument comprising a two-item depression scale (PHQ-2) and a two-item anxiety scale (GAD-2). Responses are rated on a four-point Likert scale from 0 (not at all) to 3 (nearly every day), with total scores ranging from 0 to 12. Cut-offs of 6 or greater for the total scale and 3 or greater for each subscale have been recommended as cut-offs for clinically relevant symptoms.

*Perception of Social Participation and Social Support*. Social participation was assessed using the Short Scale Measuring Perceived Social Participation (KsT-5), a five-point scale (rated on a four-point scale; 1 = disagree, 4 = agree) [20]. The responses were averaged; higher values indicate a higher level of perceived social participation. For normative values stratified by sex and age, see Berger et al. (2020) [20]. Perceived social support was assessed using the 6-item Brief Social Support Scale (BS-6). The items are rated on a 4-point Likert scale from 0 (“never”) to 3 (“always”). The responses were summed to a total score (range: 0–18), with scores ≤ 11 indicating a lack of social support [21].

#### Chronic fatigue and post-exertional malaise (PEM)

Chronic Fatigue was measured using the Chalder Fatigue Scale [22–24], which includes 11 items rated on a four-point ordinal scale (0 = less than usual, 3 = much more than usual). In the present study, the bimodal scoring procedure was applied, whereby responses coded 0 or 1 were scored as 0, and responses coded 2 or 3 were scored as 1. The resulting total score ranged from 0 to 11. A total score of 4 or more indicates severe fatigue.

PEM was assessed using the validated German version of the DSQ-PEM [25, 26]. The DSQ-PEM includes five core items specifically aimed at measuring the frequency and severity of PEM. For each of the five items, a frequency score and a severity score were summed using a 5-point Likert scale (0 = none of the time/not present, 4 = all of the time/very severe), resulting in a score ranging from 0 to 8 for each of the five items. The DSQ-PEM incorporates additional items assessing recovery time, symptom exacerbation following physical exertion, and the duration of post-exertional malaise (PEM), with a duration of ≥ 14 h being a key diagnostic criterion. A diagnosis of PEM requires the fulfillment of multiple criteria, including this prolonged recovery phase. For a comprehensive overview, see Cotler et al., 2018 and Kuczyk et al., 2025 [25, 26].

#### Medical and sociodemographic data

The data were derived from the participants’ questionnaires and from routine AOK data.

### Statistical analyses

All statistical analyses were conducted using IBM^®^ SPSS^®^ Statistics Version 29. Descriptive statistics were calculated using means, standard deviations (SDs), and ranges for continuous variables, and frequencies and percentages for categorical variables.

Two-step cluster analysis was selected for cluster formation because the algorithm is well-suited for large datasets and has the additional advantage of automatically determining the optimal number of clusters. In two-step cluster analysis, the first step involves pre-clustering observations into small subclusters, followed by a hierarchical clustering procedure that groups these subclusters into the final clusters [27, 28]. Comparative studies have identified two-step cluster analysis as a reliable method in terms of the number of subgroups identified, the accuracy of individual classification, and the reproducibility of findings across clinical and other types of datasets [27–29].

The two-step cluster analysis was performed using the 14 generated symptom impairment variables to identify groups of patients with distinct symptom patterns. The resulting clusters were subsequently compared in terms of their clinical, psychological, and sociodemographic characteristics. Kruskal–Wallis tests were used to compare continuous variables across clusters, and chi-square tests were used to compare categorical variables. Additionally, effect sizes (Eta squared, Cramér’s V, and Cohen’s omega) and 95% confidence intervals were calculated as appropriate.

As a supplementary analysis, the same analyses were then performed including the symptom presence variables.

### Sensitivity analysis

To assess cluster stability and conduct sensitivity analyses, a latent class analysis (LCA) with 100 bootstrap repetitions was performed. The optimal number of clusters was determined using the Akaike information criterion (AIC) and Bayesian (BIC) information criterion, which were supported by visual inspection of the elbow plot. Cluster agreement was assessed using the mean Adjusted Rand Index (ARI). Sensitivity analysis was performed in R (version 4.5.3).

## Results

First, we compared age, gender, and the Charlson Comorbidity Index (CCI) [30] between respondents (*n* = 2,159) and non-respondents (*n* = 18,003) and found differences with only negligible effect sizes (see Supplementary Table 1).

**Fig. 1:**
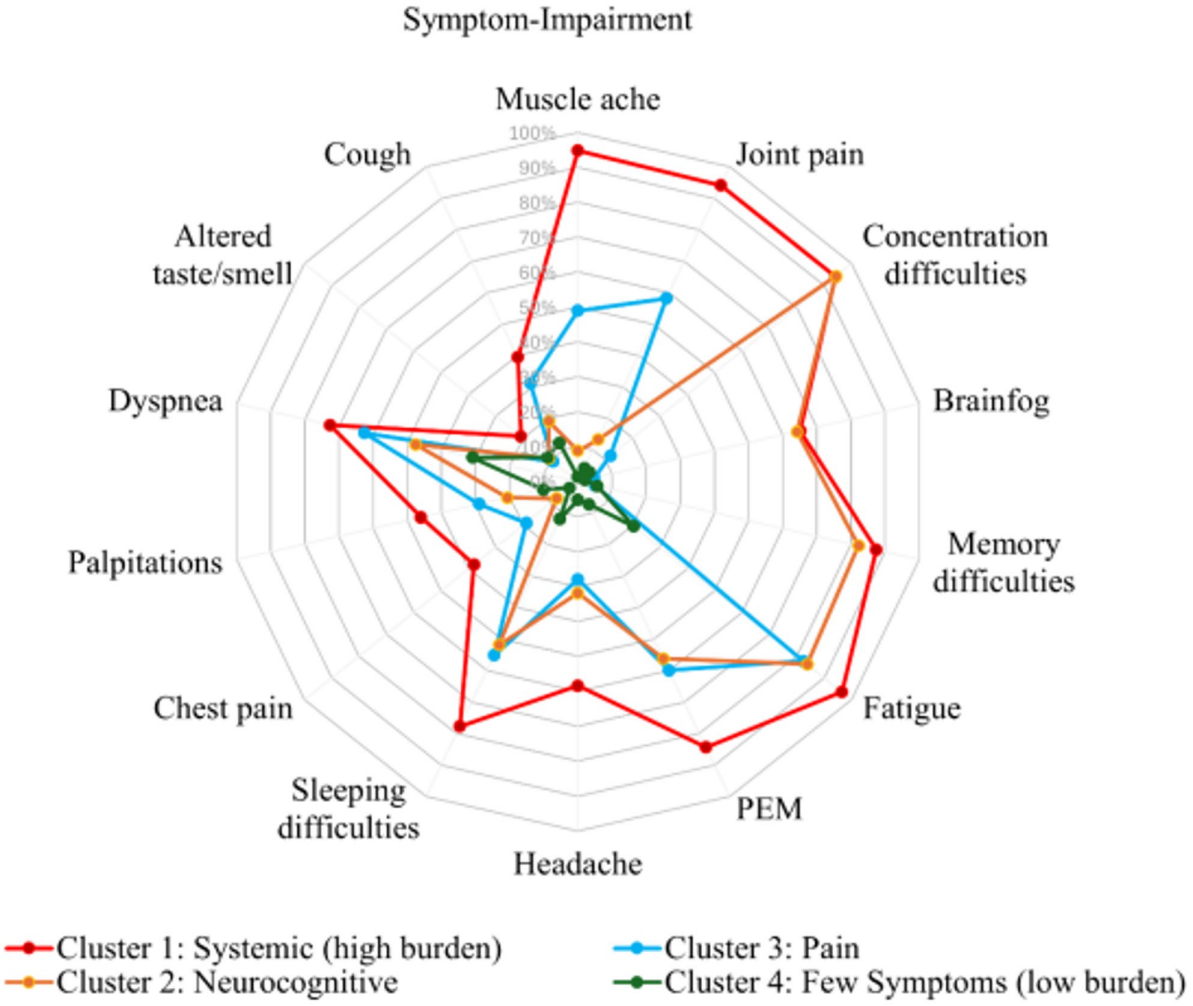
Results of the two-step cluster analysis, including the *symptom-impairment* variables: prevalence of the 14 symptoms across clusters. Cluster 1 – *Systemic (high burden)*: *N* = 461, Cluster 2 - *Neurocognitive*: *N* = 489, Cluster 3 - *Pain*: *N* = 419, Cluster 4 - *Few Symptoms (low burden)*: *N* = 304. PEM = post-exertional malaise

Among the total sample of *N* = 1,673 patients who reported ongoing PCC symptoms, 71.6% were female. The mean age was 51.3 years (SD = 12.45). Before the infection that presumably caused the PCC, 22.5% (*n* = 361) of the participants stated that they had not yet received a SARS-CoV-2 vaccination, 77.5% (*n* = 1246) had received at least one vaccination, 26.9% (*n* = 432) reported having been vaccinated twice, and 43.4% (*n* = 698) reported having received more than 2 vaccinations (data from 1607 participants available). Additional sociodemographic and clinical characteristics are presented in Table 1. The number and percentages of patients with different degrees of impairment of the 14 symptoms are summarized in Tables 2 and 3. Using the *symptom impairment* variable based on the 14 PCC symptoms, the two-step cluster analysis identified four distinct symptom clusters (see Table 4; Fig. 1). Cluster 1 *(Systemic* (high burden)) comprised 461 individuals (27.6%) and was characterized by a high prevalence of nearly all assessed symptoms. Cluster 2 *(Neurocognitive)* included 489 individuals (29.2%) and was characterized primarily by cognitive impairment, including difficulty concentrating, memory difficulties, and brain fog. Cluster 3 *(Pain)*, consisting of 419 individuals (25.0%), was distinguished by an elevated occurrence of muscle and joint pain. *Cluster 4 (Few Symptoms* (low burden), with 304 individuals (18.2%), exhibited a low prevalence of most symptoms, although fatigue (20.4%) and shortness of breath (30.9%) were the most frequently reported symptoms within this group. Overall, fatigue was the most prevalent symptom and was distributed across clusters.

**Table 1:** Sociodemographic and clinical characteristics of the total sample (*N* = 1673)

Variables	*N* = 1673	Range
Gender, female, n (%)	1198 (71.6)	
Age, years, M (SD)	51.26 (12.45)	21–93
Unemployed, yes, n (%)	397 (25.7)	
School, ≥ 12 years, n (%)	415 (28.0)	
Partnership, yes, n (%)	1201 (81.0)	
Other chronic diseases, yes, n (%)	725 (48.8)	
Time since infection, months, M (SD)	22.09 (8.48)	
PHQ-4, M (SD)	5.00 (3.28)	0–12
PHQ-4 (cut-off score ≥ 6), n (%)	271 (16.9)	
PHQ-2, M (SD)	2.63 (1.75)	0–6
PHQ-2 (cut-off score ≥ 3), n (%)	274 (17.0)	
GAD-2, M (SD)	2.37 (1.78)	0–6
GAD-2 (cut-off score ≥ 3), n (%)	232 (14.4)	
KsT-5, M (SD)	2.58 (0.67)	1–4
BS-6, M (SD)	17.48 (5.26)	6–24
CFS (total), M (SD)	8.01 (3.22)	0–11
CFS ≥ 4, n (%)	1448 (87.6)	
DSQ-PEM, M (SD)	2.91 (1.99)	0–5
PEM, cut-off ≥ 14 h, n, (%)	337 (20.6)	
Number of specialists consulted, M (SD)	2.76 (1.60)	0–8
Number of different therapies for PCC, M (SD)	2.27 (2.74)	0–23
Use of psychological therapy, n, (%)	479 (30.2)	
Use of active therapy, n, (%)	407 (25.7)	
Sick days due to PCC, M (SD)	122.76 (209.59)	0-1377
ICU due to infection n, (%)	51 (3.05)	
Improvement in PCC symptoms over time, yes, n, (%)	990 (61.5)	
Had received at least 1 vaccination prior to infection, n, (%)	1246 (77.5)	
Had received at least 1 vaccination at the time of the survey, n, (%)	1628 (97.7)	

*Notes.* PHQ-4: Patient Health Questionnaire-4. A total score of ≥ 6 is considered the cut-off for clinically relevant symptomatology.PHQ-2: Patient Health Questionnaire-2, and GAD-2: Generalized Anxiety Disorder-2. A total score of ≥ 3 is considered the cut-off for clinically relevant psychological distress. KsT-5: Short Scale for Assessing Perceived Social Participation. BS-6: Brief Social Support Scale (BS6). CFS: Chalder Fatigue Scale. DSQ-PEM: DePaul Symptom Questionnaire – Post-Exertional Malaise. Psychological therapy includes relaxation therapy, psychological counseling, and psychotherapy. Active therapy includes rehabilitative sports and functional training/sports therapy

**Table 2:** Number and percentage of participants with different degrees of *symptom impairment* of the 14 PCC symptoms

*N* = 1673	Degree of Impairment					
	Symptom not present	Not at all	Slight	Medium	Severe	Very Severe
Fatigue	382 (22.8)	2 (0.1)	27 (1.6)	360 (21.5)	550 (32.9)	352 (21.0)
PEM	714 (42.7)	1 (0.1)	21 (1.3)	241 (14.4)	414 (24.7)	282 (16.9)
Brain fog	999 (59.7)	4 (0.2)	30 (1.8)	249 (14.9)	247 (14.8)	144 (8.6)
Difficulty concentrating	678 (40.5)	-	37 (2.2)	348 (20.8)	396 (23.7)	214 (12.8)
Memory difficulties	775 (46.3)	1 (0.1)	54 (3.2)	301 (18.0)	357 (21.3)	185 (11.1)
Chest Pain	1329 (79.4)	4 (0.2)	39 (2.3)	168 (10.0)	101 (6.0)	32 (1.9)
Joint pain	876 (52.4)	3 (0.2)	38 (2.3)	241 (14.4)	310 (18.5)	205 (12.3)
Muscle pain/cramps	940 (56.2)	1 (0.1)	43 (2.6)	221 (13.2)	268 (16.0)	200 (12.0)
Headache	1074 (64.2)		41 (2.5)	204 (12.2)	226 (13.5)	128 (7.7)
Heart palpitations	1141 (68.2)	5 (0.3)	62 (3.7)	236 (14.1)	165 (9.9)	64 (3.8)
Shortness of breath	692 (41.4)	3 (0.2)	54 (3.2)	341 (20.4)	383 (22.9)	200 (12.0)
Cough	1170 (69.9)	1 (0.1)	58 (3.5)	203 (12.1)	167 (10.0)	74 (4.4)
Sleep disorder	768 (45.9)	-	26 (1.6)	236 (14.1)	363 (21.7)	280 (16.7)
Loss of smell/taste	1420 (84.9)	2 (0.1)	31 (1.9)	74 (4.4)	68 (4.1)	78 (4.7)

*Notes*. PEM = post-exertional malaise

**Table 3:** Percent of participants with moderate, severe, or very severe impairment based on the 14 PCC symptoms and mean impairment rating (1–5) (*N* = 1673)

	Percent			Mean impairment		
	Total sample *N* = 1673	Female *N* = 1198	Male *N* = 475	Total sample *N* = 1673	Female *N* = 1198	Male *N* = 475
Fatigue	77.2	77.4	77.5	3.95	3.97	3.89
PEM	57.3	56.8	59.2	4.00	4.00	4.00
Brain fog	40.3	41.2	38.3	3.74	3.75	3.70
Difficulty concentrating	59.5	61.2	55.8	3.79	3.82	3.71
Memory difficulties	53.7	55.3	50.1	3.75	3.76	3.72
Chest Pain	20.6	20.4	21.5	3.34	3.32	3.40
Joint pain	47.6	48.4	46.5	3.85	3.86	3.81
Muscle pain/cramps	43.8	44.8	41.9	3.85	3.89	3.74
Headache	35.8	38.0	30.7	3.74	3.80	3.53
Heart palpitations	31.8	34.4	25.9	3.42	3.44	3.34
Shortness of breath	58.6	57.7	61.5	3.74	3.68	3.88
Cough	30.1	29.4	32.2	3.51	3.53	3.44
Sleep disorder	54.1	55.4	51.6	3.99	4.01	3.94
Loss of smell/taste	15.1	16.0	13.1	3.75	3.71	3.85

*Notes*: For all symptoms reported as currently present, the degree of impairment was assessed using the question: *“How severely do the following currently existing symptoms impair you?”* Responses were given on a five-point Likert scale (not at all, slightly, moderately, severely, very severely)

**Table 4:** Four clusters resulting from the two-step cluster analysis based on the *symptom-impairment* variables, with the frequency n (%) of the 14 symptoms in the total sample and within each cluster. Symptoms are sorted in descending order of importance for cluster formation

Symptom Variables *n* (%)		Cluster			
	Total *n* = 1673	Systemic (high burden) *n* = 461	Neuro-cognitive *n* = 489	Pain *n* = 419	Few Symptoms (low burden) *n* = 304
Difficulty concentrating	958 (57.3)	434 (94.1)	461 (94.3)	50 (11.9)	13 (4.3)
Memory difficulties	843 (50.4)	403 (87.4)	402 (82.2)	21 (5.0)	17 (5.6)
Muscle pain/cramps	689 (41.2)	437 (94.8)	43 (8.8)	205 (48.9)	4 (1.3)
Joint Pain	756 (45.2)	434 (94.1)	65 (13.3)	244 (58.2)	13 (4.3)
Brain fog	640 (38.3)	300 (65.1)	314 (64.2)	20 (4.8)	6 (2.0)
Fatigue	1262 (75.4)	445 (96.5)	410 (83.8)	345 (82.3)	62 (20.4)
PEM	937 (56.0)	389 (84.4)	275 (56.2)	251 (59.9)	22 (7.2)
Sleep disorder	879 (52.5)	358 (77.7)	254 (51.9)	231 (55.1)	36 (11.8)
Headache	558 (33.4)	269 (58.4)	156 (31.9)	117 (27.9)	16 (5.3)
Chest Pain	301 (18.0)	175 (38.0)	38 (7.8)	79 (18.9)	9 (3.0)
Shortness of breath	924 (55.2)	335 (72.7)	233 (47.6)	262 (62.5)	94 (30.9)
Heart Palpitations	465 (27.8)	212 (46.0)	101 (20.7)	121 (28.9)	31 (10.2)
Cough	444 (26.5)	182 (39.5)	94 (19.2)	131 (31.3)	37 (12.2)
Loss of smell/taste	220 (13.2)	96 (20.8)	52 (10.6)	38 (9.1)	34 (11.2)

The FIT indices, the importance of the individual symptoms, and the cluster quality (which was fair) are depicted in Supplementary Tables 2 and Supplementary Figs. 1 and 2.

Using the presence or absence of the 14 PCC symptoms, the cluster structure closely mirrored that of the *symptom impairment* analysis, yielding: Cluster 1 *(Systemic* (high burden)) (*n* = 426; 25.5%), Cluster 2 *(Neurocognitive)* (*n* = 447; 26.7%), Cluster 3 *(“Pain”)* (*n* = 369; 22.1%), and Cluster 4 *(Few Symptoms* (low burden)) (*n* = 431; 25.8%) (see Supplementary Tables 3–4 and Supplementary Figs. 3–5).

## Comparison of clusters on sociodemographic, psychological, and clinical variables

Participants in the *Systemic* (high burden) *cluster* exhibited the highest scores on the anxiety and depression scales (PHQ-4, PHQ-2, GAD-2), with group comparisons across all clusters demonstrating large effect sizes. Concerning sociodemographic characteristics, only minor effects were noted for age, employment status and duration of education. The largest proportion of unemployed patients was identified in the *Systemic* (high burden) *cluster*. No significant differences were observed among the groups with respect to gender and relationship status.

Patients classified within the *Systemic* (high burden) *cluster* also demonstrated the highest scores on the Chalder Fatigue Scale and the DSQ-PEM. Comparative analyses across all clusters indicated large effect sizes. The utilization of therapeutic services (including the total number of therapies, psychological therapy, and active therapy) was also most pronounced within the *Systemic* (high burden) *cluster*; small to moderate effect sizes were observed across cluster comparisons.

No significant differences were observed between the clusters regarding the number of inpatient or intensive care treatments attributable to PCC.

Although group differences regarding vaccination status were observed, these were associated with only very small effect sizes. For detailed statistical results, please refer to Tables 5 and 6 and Supplementary Tables 5–7.

**Table 5:** Comparison of the *symptom-impairment* clusters regarding sociodemographic, clinical, and psychological continuous variables

Variables	Cluster				Kruskal-Wallis-test (df = 3 H, *p*, η² 95%-CI)
M (SD)	Systemic (high burden) *n* = 461	Neurocognitive *n* = 489	Pain *n* = 419	Few Symptoms (low burden) *n* = 304	
Age (years)	52.59 (10.66)	49.15 (12.63)	52.73 (12.60)	50.62 (13.91)	H = 21.48 *p* < .001 η² = 0.01 [0.00-0.03]
PHQ-4	6.62 (3.22)	5.88 (3.04)	4.00 (2.65)	2.38 (2.41)	H = 380.53 *p* < .001 η² = 0.24 [0.20–0.28]
PHQ-2	3.48 (1.71)	3.08 (1.61)	2.18 (1.45)	1.19 (1.27)	H = 382.05 *p* < .001 η² = 0.24 [0.20–0.27]
GAD-2	3.14 (1.75)	2.81 (1.72)	1.82 (1.48)	1.19 (1.43)	H = 291.71 *p* < .001 η² = 0.18 [0.16–0.21]
BS-6	16.83 (5.09)	17.36 (5.11)	18.00 (5.17)	18.03 (5.77)	H = 18.22 *p* < .001 η² = 0.01 [0.00-0.03]
KsT-5	11.68 (3.32)	12.57 (3.28)	13.52 (3.06)	14.61 (3.10)	H = 133.42 *p* < .001 η² = 0.09 [0.06–0.12]
CFS (total)	10.18 (1.58)	9.28 (2.08)	7.04 (2.68)	3.94 (3.04)	H = 765.09 *p* < .001 η² = 0.46 [0.42–0.50]
CFS (physical)	17.17 (2.95)	15.07 (3.30)	13.78 (3.40)	9.40 (3.44)	H = 619.51 *p* < .001 η² = 0.37 [0.34–0.40]
CFS (mental)	8.98 (2.03)	8.53 (2.11)	5.38 (2.09)	4.66 (2.04)	H = 753.79 *p* < .001 η² = 0.46 [0.42–0.49]
DSQ-PEM	4.29 (1.29)	3.22 (1.81)	2.51 (1.80)	0.76 (1.31)	H = 588.33 *p* < .001 η² = 0.36 [0.33–0.40]
Number of different therapies for PCC	3.10 (3.12)	2.32 (2.59)	2.04 (2.60)	1.21 (2.04)	H = 86.24 *p* < .001 η² = 0.05 [0.03–0.07]
Number of specialists consulted	3.35 (1.73)	2.86 (1.57)	2.56 (1.46)	1.92 (1.15)	H = 139.10 *p* < .001 η² = 0.09 [0.06–0.11]
Time since infection, months	23.29 (9.06)	22.40 (8.62)	21.14 (7.87)	21.06 (7.90)	H = 18.94 *p* < .001 η² = 0.01 [0.00-0.03]
Sick days due to PCC	201.88 (263.93)	133.94 (223.74)	94.78 (163.52)	35.12 (76.30)	H = 133.34 *p* < .001 η² = 0.12 [0.08–0.15]

*Notes.* PHQ-4: Patient Health Questionnaire-4; PHQ-2: Patient Health Questionnaire-2; GAD-2: Generalized Anxiety Disorder-2; BS-6: Brief Social Support Scale. KsT-5: Short Scale Measuring Perceived Social Participation; CFS: Chalder Fatigue-Scale; DSQ-PEM: DePaul Symptom Questionnaire – Post-Exertional Malaise

**Table 6:** Comparison of the *symptom-impairment* clusters regarding sociodemographic, clinical, and psychological categorical variables

Variables	Cluster				Chi-square test (df = 3 χ^2^, *p*, V, 95%-CI)
n (%)	Systemic (high burden) *n* = 461	Neurocognitive *n* = 489	Pain *n* = 419	Few Symptoms (low burden) *n* = 304	
Gender, female	342 (74.2)	358 (73.2)	286 (68.3)	212 (69.7)	χ^2^ = 4.96 *p* = .175
Unemployed	143 (33.4)	102 (22.2)	89 (22.8)	63 (23.5)	χ^2^ = 18.61 *p* < .001 V = 0.11 [0.05–0.15]
School ≥ 12 years.	99 (24.0)	137 (31.1)	94 (25.3)	85 (33.5)	χ^2^ = 10.51 *p* = .015 V = 0.08 [0.02–0.13]
Partnership	330 (79.5)	350 (79.2)	317 (85.2)	204 (80.3)	χ^2^ = 5.91 *p* = .116
Other chronic diseases	244 (58.8)	199 (45.0)	184 (49.2)	98 (38.4)	χ^2^ = 30.11 *p* < .001 V = 0.14 [0.09–0.19]
PHQ- 4 ≥ 6	134 (49.4)	103 (38.0)	27 (10.0)	7 (2.6)	χ^2^ = 233.67 *p* < .001 V = 0.27 [0.23–0.30]
DSQ-PEM: Dead, heavy feeling after starting to exercise	428 (94.1)	351 (72.4)	279 (68.0)	65 (22.8)	χ^2^ = 418.31 *p* < .001 V = 0.51 [0.46–0.55]
DSQ-PEM: Next day soreness or fatigue after non-strenuous, everyday activities	388 (85.3)	274 (56.6)	195 (47.6)	30 (10.5)	χ^2^ = 404.51 *p* < .001 V = 0.50 [0.45–0.54]
DSQ-PEM: Mentally tired after the slightest effort	375 (82.4)	346 (71.3)	132 (32.2)	33 (11.6)	χ^2^ = 491.86 *p* < .001 V = 0.55 [0.50–0.60]
DSQ-PEM: A minimum exercise makes you physically tired	385 (84.6)	296 (61.0)	208 (50.7)	46 (16.1)	χ^2^ = 345.84 *p* < .001 V = 0.46 [0.41–0.51]
DSQ-PEM: Physically drained or sick after mild activity	377 (82.9)	297 (61.2)	217 (52.9)	43 (15.1)	χ^2^ = 334.94 *p* < .001 V = 0.45 [0.40–0.50]
PEM recovery time ≥ 14 h	168 (49.9)	89 (26.4)	64 (19.0)	16 (4.7)	χ^2^ = 121.00 *p* < .001 V = 0.27 [0.22–0.32]
CFS ≥ 4	452 (31.2)	474 (32.7)	372 (25.7)	150 (10.4)	χ^2^ = 462.71 *p* < .001 V = 0.53 [0.48–0.58]
Use of psychological therapy	180 (40.9)	160 (34.2)	98 (24.6)	41 (14.7)	χ^2^ = 64.95 *p* < .001 V = 0.20 [0.15–0.25]
Use of active therapy	145 (33.0)	127 (27.1)	92 (23.1)	43 (15.5)	χ^2^ = 29.35 *p* < .001 V = 0.14 [0.08–0.18]
Inpatient admission due to post-COVID	48 (10.7)	46 (9.6)	38 (9.4)	15 (5.3)	χ^2^ = 6.65 *p* = .084
ICU due to infection	19 (4.1)	11 (2.2)	12 (2.9)	9 (3.0)	χ^2^ = 5.50 *p* = .139
Improvement in PCC symptoms over time, yes	192 (43.3)	259 (64.0)	310 (65.0)	229 (80.6)	χ^2^ = 109.12 *p* < .001 V = 0.26 [0.21–0.31]
Had received at least 1 SARS-CoV-2 vaccination prior to infection	326 (74.1)	328 (81.0)	356 (75.9)	236 (80.5)	χ^2^ = 8.01 *p* = .046 V = 0.07 [0.00-0.11]
Had received at least 1 SARS-CoV-2 vaccination at the time of the survey	448 (97.8)	410 (97.9)	474 (97.3)	296 (98.0)	χ^2^ = 0.50 *p* = .919

*Notes.* CFS: Chalder-Fatigue-Scale; DSQ-PEM: DePaul Symptom Questionnaire – Post-Exertional Malaise. Psychological therapy includes relaxation therapy, psychological counseling, and psychotherapy. Active therapy includes rehabilitative sports and functional training/sports therapy

### Sensitivity analysis

The optimal number of clusters selected was 4. Assessment of cluster stability using a latent class analysis with 4 classes and 100 bootstrap repetitions yielded a mean ARI of 0.809 (SD = 0.121). This is considered excellent agreement between the original cluster assignments and those derived from the bootstrap samples, suggesting a stable and reliable cluster solution.

## Discussion

This study aimed to identify symptom clusters in the long-term course after SARS-CoV-2 infection in a large sample of patients with PCC. Data were obtained from the largest health insurance providers in Lower Saxony, ensuring that all patients included had a medically confirmed PCC diagnosis. The identified clusters were subsequently compared with respect to sociodemographic, clinical, and psychological characteristics.

The following four *symptom impairment* clusters emerged: *Systemic (*high burden) *(Cluster 1)*,* Neurocognitive (Cluster 2)*,* Pain (Cluster 3)*,* and Few Symptoms* (low burden) *(Cluster 4). Clusters 1 and 4* differed primarily in overall symptom severity, with this distinction between high- and low-symptom subgroups corresponding to previous findings from symptom-based cluster analyses in ME/CFS [31–34]. Participants in the *“Few Symptom”* cluster had fewer comorbidities overall and showed lower psychological distress.

Beyond these general severity clusters, two more specific symptom clusters emerged. Participants in *Cluster 2*, which was the largest cluster, exhibited difficulty concentrating, brain fog, and memory difficulties as their leading symptoms, which is why this cluster was described as a “*Neurocognitive”* cluster. Similar neurocognitive subgroups have been identified in earlier studies, e.g., by Grafström et al. (2025) [9] six months after infection or by Moniz et al. (2024) [35] at 9- and 12-month post-infection. In our study, the mean time since infection was 22 months, suggesting that this cluster represents a stable, long-term pattern.

*Cluster 3* was defined by the predominance of muscle pain/cramps and joint pain, and was therefore defined as a *“Pain”* cluster, consistent with previous reports of pain-focused subgroups in ME/CFS and PCC [31, 32, 36].

As expected, substantial symptom overlap occurred between the clusters. Fatigue and PEM central symptoms of ME/CFS- were present in all clusters, although only 5 to 50% in each cluster reported that post-exertional malaise lasted more than 14 h after activities.

The analysis incorporating *symptom presence* variables yielded essentially the same clusters as described earlier, but with a different distribution/varying group size. In particular, the low-symptom cluster was significantly larger in the *symptom presence* cluster analysis. A supplementary exploratory examination of individuals whose cluster membership differed between the two cluster analyses revealed the following pattern: individuals with a low overall symptom burden were more frequently assigned to the low-symptom cluster when the presence variables were taken into account. However, if these individuals reported at least moderate to severe impairment in a single symptom of a symptom area (pain, neurocognitive), they were more likely to be assigned to a symptom-specific cluster when the impairment variables were included.

Notably, the survey referred to an early stage of the COVID-19 pandemic, when vaccinations may not yet have been widely available or available for only a short period. Thus, the non-vaccination rate in our sample was still high, with 22.5% not having received any vaccination prior to the infection that presumably caused the PCC. Participants in the *Systemic* (high burden) cluster had the highest non-vaccination rate (25.9%). In comparison, among participants who reported no longer having any PCC symptoms, only 9.7% (*N* = 45) had not been vaccinated against SARS-CoV-2 prior to infection. These results suggest that vaccination may protect against the development or persistence of PCC symptoms. Several cohort studies and meta-analyses have reported a lower incidence of PCC among individuals who received at least one dose of a COVID-19 vaccine [37, 38].

### Hypotheses and future directions

One study attempted to define clusters based on biological changes rather than subjective symptoms. Asprusten et al. (2021) [39] identified clusters in adolescents with ME/CFS using biomarkers across five domains (endocrine, inflammatory, cardiovascular, pressure pain threshold, and cognitive).

However, substantial overlap between clusters limited the identification of distinct pathophysiological subtypes.

While biomarker-based clustering in ME/CFS has proven challenging, convergent evidence from mechanistic studies in PCC - including viral persistence, autoimmunity, immune exhaustion leading to latent virus reactivation, and severe metabolic disruption - now suggests pathway-specific biological explanations for symptom-based clusters.

The present study is a cross-sectional symptom survey based entirely on self-report; no biomarkers, imaging data, biological samples, or objective clinical outcomes were collected. The cluster-to-mechanism mappings discussed below are therefore presented as illustrative, testable hypotheses for future investigation, and are not findings of the present analysis. Importantly, this study was primarily clinical and not designed to directly investigate the underlying biological mechanisms of PCC. Therefore, the following attempts to translate our clinical clusters into potential biological pathways are highly speculative and should not be interpreted as definitive conclusions. However, integrating our symptom-based findings with current mechanistic research allows us to generate testable hypotheses regarding the pathophysiology of different PCC phenotypes [40].

Cluster 1 (*Systemic* (high burden)) might represent a state of persistent viral antigen-driven pathology coupled with severe metabolic failure. The multi-organ involvement and heavy symptom burden align with recent evidence that SARS-CoV-2 RNA and antigens can persist in tissue reservoirs (such as the gut and nervous system) for over a year [41]. This viral persistence continuously stimulates the immune system, causing widespread T cell activation GADie [42], sustained activation of JAK-STAT and IL-6 pathways, and immunothrombotic cascades that persist months post-infection [10, 43]. Critically, this chronic inflammation does not require active viral replication. We recently demonstrated in animal models that intravenous administration of the SARS-CoV-2 spike (S1) protein alone induces widespread neuroinflammation, glial activation, and alpha-synuclein accumulation [44], supporting a “protein-as-pathogen” mechanism in which circulating viral components drive multisystem injury.

Over time, this chronic antigenic stimulation can drive profound immune exhaustion, marked by persistent lymphopenia [45], which is a major, well-documented trigger for the reactivation of latent viruses [46]. Crucially, the reactivation of Epstein-Barr virus (EBV) has recently been found at significantly higher rates in PCC patients suffering from persistent fatigue and post-exertional malaise [47]. Furthermore, the reactivation of Herpes Zoster following COVID-19 acts as a strong clinical indicator of this underlying systemic immune dysregulation, substantially increasing the risk for severe downstream autoimmune complications [48]. The profound physical exhaustion defining this cluster is also likely driven by severe disruptions in amino acid metabolism, oxidative stress [49], and persistent mitochondrial dysfunction, marked by specific urine metabolite disruptions [50] and highly predictive metabolic signatures (e.g., elevated alpha-ketoglutarate and reduced creatine) which tightly correlate with physical impairment [51].

The symptom profile of Cluster 2 (*Neurocognitive*) shows similarities to phenotypes that have been associated, in independent studies, with early neurodegenerative and neuroinflammatory processes. Structurally, patients in this cluster might exhibit hippocampal iron deposition and cortical thinning on neuroimaging, accompanied by blood biomarkers indicating astrocytic damage [52]. The preclinical work of some co-authors of this manuscript provides a cellular mechanism: SARS-CoV-2 infection initiates persistent accumulation of alpha-synuclein and Tau in hippocampal and cortical regions, establishing a proteinopathy that outlasts viral clearance [53]. The specific symptom of “brain fog” may be mechanistically linked to dysfunction of parvalbumin-positive (PV+) interneurons, which generate the gamma oscillations critical for cognitive focus. These interneurons are significantly altered in post-COVID-19 models [54], potentially explaining the attentional deficits that define this cluster. The female predominance observed in this cluster aligns with clinical evidence of heightened inflammatory T-cell responses in women [55] and is directly paralleled in animal models, where females exhibit more severe biphasic neuroinflammatory responses, greater alpha-synuclein burden, and more pronounced PV+ interneuron dysfunction than males [44, 54]. This specific clinical presentation is now also strongly supported by recent proteomic discoveries identifying highly accurate diagnostic blood biomarkers -such as elevated C5a, TGFβ1, and Gliomedin - specifically for neurologic manifestations of long COVID-19 (Neuro-PASC) [56].

Cluster 3 (*Pain*) appears mechanistically distinct from broad systemic inflammation, possibly driven instead by targeted autoimmune and peripheral nervous system pathology.

Passive transfer experiments in independent samples have shown that IgG from patients with long COVID-19 pain can induce small-fiber neuropathy and mechanical hypersensitivity in mice by directly binding to sensory neurons [57, 58]. Additionally, viral peptides can directly activate spinal TLR4 signaling pathways, triggering central sensitization and sustained nociception [59]. Together, these mechanisms suggest a candidate biological framework, which is to be tested in future work, that could plausibly underlie the chronic pain phenotype observed in this cluster.

Cluster 4 (*Few Symptoms* (low burden)) may represent successful immune resolution. This favorable outcome may be mediated by tolerogenic immune profiles, particularly the emergence of IgG4 antibodies, which prevent the chronic inflammatory and autoimmune cascades observed in the more severe clusters [60, 61].

In summary, we propose, strictly as hypotheses for future testing, that the four symptom clusters identified here may correspond to phenotypes with distinct mechanisms: systemic viral persistence, latent virus reactivation, and amino acid/metabolic disruption (Cluster 1); localized neurodegeneration and neuroinflammation with possible sex-specific vulnerability (Cluster 2); peripheral autoimmunity and central sensitization (Cluster 3); and successful immune regulation (Cluster 4). Whether this is the case can only be established by future biomarker, imaging, and longitudinal studies. Empirically testing the cluster-mechanism correspondences proposed above is therefore a necessary prerequisite to any precision-medicine application [41], in which cluster assignment might eventually guide targeted immunomodulatory, neuroprotective, or pain-specific interventions.

### Strengths and limitations

The present study has several strengths. First, its large sample size provides greater robustness than previous studies with smaller sample sizes. Second, patient selection was based on physician confirmed PCC diagnoses. Third, the assessment of both symptom prevalence and impairment facilitated a more realistic and nuanced characterization of symptom burden and, in turn, of cluster differentiation.

Importantly, our study has certain limitations that must be considered. We had a 10.7% response rate for those who were mailed the questionnaire, which might have introduced any kind of bias. Nevertheless, to more accurately delineate the latter, we conducted a comparison of age, gender, and the CCI between respondents and non-respondents, and observed differences with only negligible effect sizes.

Even though our *Few Symptoms* (low burden) cluster was the smallest, individuals with a very high neurocognitive symptom burden might have been less likely to participate. Conversely, people whose symptoms had subsided in the meantime may also have been less willing to participate. Overall, selection bias in both directions can therefore be assumed, suggesting that predominantly patients with moderate symptoms were included, although no definitive statement can be made on this. Additionally, similar to earlier studies, the participants were predominantly females (71%), although it has been recognized that the prevalence of PCC is higher in women than in men.

Furthermore, participants were identified via the ICD-10 code U09.9 from routine health insurance data. The thoroughness of the clinical assessment and the coding practices most likely varied between physicians. We only included people who received the diagnosis in 2022 and we do have data on hospital and ICU admission during the COVID-19 infection as a proxy for severity. However, we do not have confirmed data on the timing of the infection, the virus variant, and prior re-infections and, thus, cannot exclude sample heterogeneity and increased risk of spurious cluster formation.

The cluster quality was fair, and there was a high overlap of symptom prevalence and symptom impairment between clusters. We lack pre-infection data on symptom burden, and we could not provide longitudinal data. We did not correct for multiple testing due to the explorative nature of this research; however, the differences between clusters were big, with p-values well below the threshold of significance and medium to large effect sizes.

Finally, and most fundamentally, the present study did not include any biomarkers, imaging, biological samples, or objective clinical outcomes. We therefore cannot test whether the identified symptom clusters correspond to biologically distinct subgroups, and the mechanistic interpretations offered in the section above are presented exclusively as hypotheses for future investigation.

Overall, this study’s findings extend the current literature on symptom-based cluster analyses in PCC by demonstrating the presence of both general clusters of symptom burden and specific subgroups, particularly pain and neurocognitive clusters. Fatigue and PEM in patients occurred as central symptoms across nearly all clusters, underscoring the significant overlap between PCC and ME/CFS symptomatology. Classifying PCC patients by severity and characteristic symptom patterns could help develop more targeted, individually tailored therapeutic approaches. Future research should aim to define biologically defined subgroups with greater precision to clarify the underlying pathophysiological mechanisms of different symptom clusters.

## Supplementary Information

Below is the link to the electronic supplementary material.

Supplementary Material 1

## Data Availability

The datasets generated and/or analyzed during the current study are available from the corresponding author upon reasonable request.
