# Supplementary material for: Symptom-based clusters in people with post-COVID-19 condition (PCC)
Source: J Transl Med. 2026 Jul 11;24:892. doi: 10.1186/s12967-026-08582-4 (PMC13362145; doi:10.1186/s12967-026-08582-4)
Supplement: Supplementary file 1 — Supplementary Material 1 [file 12967_2026_8582_MOESM1_ESM.docx]

**Symptom-based clusters in people with post-COVID-19 condition (PCC)**

**Supplement**

**Summary**

**Supplementary Table 1**. Comparison between Respondents and Non-respondents.

**Supplementary Table 2.** FIT indices of the two-step cluster analysis, including the 14 *symptom-impairment* variables (included: only patients who are currently still affected).

**Supplementary Table 3.** The four clusters resulting from the two-step cluster analysis based on the *symptom-presence* variables are shown, with the frequency n (%) of the 14 symptoms in the total sample and within each cluster. The symptoms are sorted in descending order of importance for cluster formation.

**Supplementary Table 4.** FIT indices of the two-step cluster analysis, including the 14 *symptom-presence* variables (including only patients currently still affected).

**Supplementary Table 5**. Comparison of the *symptom-presence* clusters regarding sociodemographic, clinical, and psychological continuous variables.

**Supplementary Table 6.** Comparison of the *symptom-presence* clusters regarding sociodemographic, clinical, and psychological categorical variables.

**Supplementary Table 7.** Comparison of the *symptom-impairment* clusters regarding comorbid chronic conditions.

**Supplementary Figure 1.** Results of the two-step cluster analysis including the symptom-impairment variables: graphical representation of predictor importance across the 14 PCC symptoms.

**Supplementary Figure 2.** Results of the two-step cluster analysis, including the symptom-impairment variables: graphical representation of cluster quality

**Supplementary Figure 3.** Results of the two-step cluster analysis, including the *symptom-presence* variables: prevalence of the 14 symptoms across clusters. Cluster 1 – Systemic (high burden): N = 426; Cluster 2 – *Neurocognitive*: N = 447; Cluster 3 – *Pain*: N = 369; Cluster 4 – *Few Symptoms* (low burden): N = 431.

**Supplementary Figure 4.** Results of the two-step cluster analysis, including the symptom-presence variables: graphical representation of predictor importance across the 14 symptom variables.

**Supplementary Figure 5.** Results of the two-step cluster analysis, including the symptom-presence variables: graphical representation of cluster quality.

|  | **Respondents** | | **Non-respondents** | | **Chi-square test**  (χ^2,^ p, ω) | |
| --- | --- | --- | --- | --- | --- | --- |
|  | n | % | n | % | n | % |
|  | 2159 | 100 | 18003 | 100 | 20162 | 100 |
| **Age Group** |  | | | | | |
| 18-35 | 384 | 17.8 | 4887 | 27.1 | χ^2^ **=** 213.63,  p < .001,  ω = 0.10 | |
| 36-50 | 717 | 33.2 | 5083 | 28.2 |  |  |
| 51-65 | 935 | 43.3 | 5875 | 32.6 |  |  |
| > 65 | 123 | 5.7 | 2158 | 12 |  |  |
|  | | | | | | |
| **Gender** |  | | | | | |
| Male | 623 | 28.9 | 6879 | 38.2 | χ^2^ **=** 72.20,  p < .001,  ω = 0.06 | |
| Female | 1536 | 71.1 | 11124 | 61.8 |  |  |
|  | | | | | | |
| **CCI** | | | | | | |
| 0 | 1278 | 59.2 | 10829 | 60.2 | χ^2^ **=** 14.14,  p < .001,  ω = 0.03 | |
| 1-2 | 710 | 32.9 | 5499 | 30.5 |  |  |
| 3-4 | 142 | 6.6 | 1233 | 6.8 |  |  |
| ≥5 | 29 | 1.3 | 442 | 2.5 |  |  |

**Supplementary Table 1**. Comparison between Respondents and Non-respondents.

*Notes.* CCI = Charlson Comorbidity Index. The CCI was categorized as low (1–2), intermediate (3–4), or high (≥5). Higher scores indicate a greater burden of comorbidities.

**Supplementary Table 2.** FIT indices of the two-step cluster analysis, including the 14 *symptom-impairment* variables (included: only patients who are currently still affected).

| Number of Clusters | Schwarz’s Bayesian Criterion (BIC) | BIC Change ^a^ | Ratio of BIC Changes ^b^ | Ratio of Distance Measures ^c^ |
| --- | --- | --- | --- | --- |
| **1** | **29203.18** |  |  |  |
| **2** | **25320.01** | **-3883.17** | **1.00** | **1.96** |
| **3** | **23384.35** | **-1935.66** | **0.50** | **1.63** |
| **4** | **22233.06** | **-1151.29** | **0.30** | **2.01** |
| 5 | 21713.12 | -519.94 | 0.13 | 1.12 |
| 6 | 21257.61 | -455.51 | 0.12 | 1.32 |
| 7 | 20936.18 | -321.43 | 0.08 | 1.11 |
| 8 | 20655.88 | -280.30 | 0.07 | 1.02 |
| 9 | 20383.02 | -272.85 | 0.07 | 1.04 |
| 10 | 20123.31 | -259.71 | 0.07 | 1.05 |
| 11 | 19881.90 | -241.41 | 0.06 | 1.19 |
| 12 | 19695.40 | -186.50 | 0.05 | 1.02 |
| 13 | 19514.09 | -181.31 | 0.05 | 1.02 |
| 14 | 19338.08 | -176.01 | 0.05 | 1.08 |
| 15 | 19182.69 | -155.40 | 0.04 | 1.14 |

*Notes:* ^a^ The changes are from the previous number of clusters in the table. ^b^ The ratios of changes are relative to the change for the two-cluster solution. ^c^ The ratios of distance measures are based on the current number of clusters against the previous number of clusters. The cluster solution that best fits the data according to the fit indices is highlighted in bold.

**Supplementary Figure 1.** Results of the two-step cluster analysis including the symptom-impairment variables: graphical representation of predictor importance across the 14 PCC symptoms.


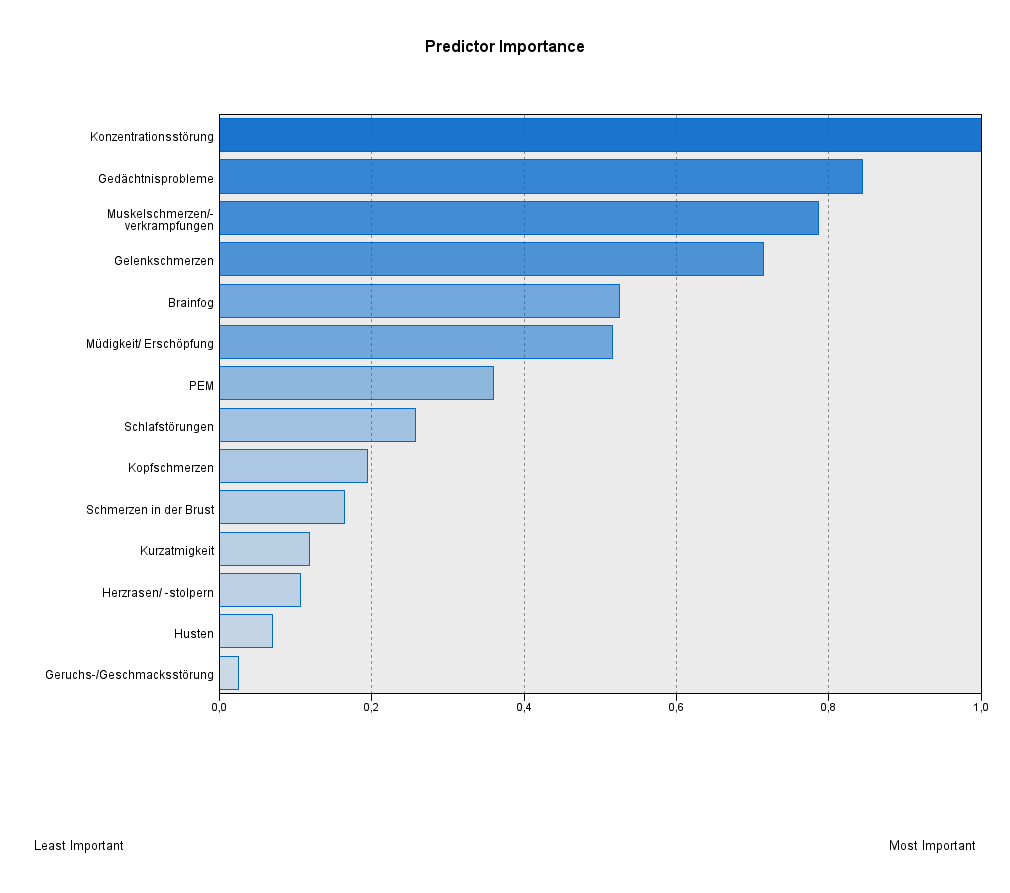


Concentration difficulties

Memory difficulties

Muscle pain/cramps

Joint pain

Brain fog

Fatigue/exhaustion

PEM

Sleep disorders

Headaches

Chest pain

Shortness of breath

Heart palpitations

Cough

Loss of smell/taste

**Supplementary Figure 2.** Results of the two-step cluster analysis, including the symptom-impairment variables: graphical representation of cluster quality


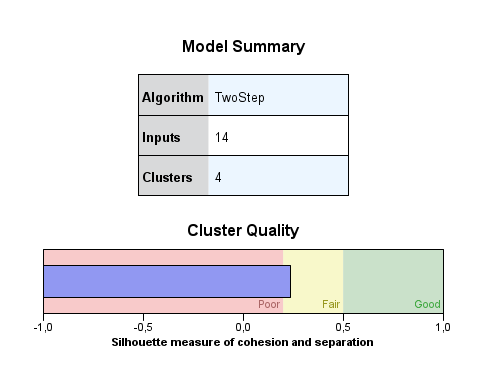


**Supplementary Table 3.** The four clusters resulting from the two-step cluster analysis based on the *symptom-presence* variables are shown, with the frequency n (%) of the 14 symptoms in the total sample and within each cluster. The symptoms are sorted in descending order of importance for cluster formation.

|  |  | Cluster | | | |
| --- | --- | --- | --- | --- | --- |
| Symptom  Variables n (%)  (1-14) | Total  N = 1673 | Systemic (high burden)  N = 426 | Neurocognitive  N =447 | Pain  N = 369 | Few  Symptoms  (low burden)  N = 431 |
| Muscle pain/cramps | 736 (44.0) | 371 (87.1) | 30 (6.7) | 323 (87.5) | 12 (2.8) |
| Difficulty concentrating | 998 (59.7) | 414 (97.2) | 423 (94.6) | 148 (40.1) | 13 (3.0) |
| Joint Pain | 801 (47.9) | 376 (88.3) | 47 (10.5) | 341 (92.4) | 37 (8.6) |
| Brain fog | 676 (40.4) | 366 (85.9) | 276 (61.7) | 27 (7.3) | 7 (1.6) |
| Memory difficulties | 901 (53.9) | 408 (95.8) | 350 (78.3) | 114 (30.9) | 29 (6.7) |
| Fatigue | 1295 (77.4) | 417 (97.9) | 373 (83.4) | 312 (84.6) | 193 (44.8) |
| PEM | 962 (57.5) | 370 (86.9) | 229 (51.2) | 238 (64.5) | 125 (29.0) |
| Headache | 601 (35.9) | 279 (65.5) | 134 (30.0) | 128 (34.7) | 60 (13.9) |
| Sleep disorder | 909 (79.1) | 337 (79.1) | 219 (49.0) | 233 (63.1) | 120 (27.8) |
| Chest pain | 346 (20.7) | 179 (42.0) | 40 (8.9) | 86 (23.3) | 41 (9.5) |
| Heart palpitations | 535 (32.0) | 224 (52.6) | 103 (23.0) | 125 (33.9) | 83 (19.3) |
| Shortness of breath | 983 (58.8) | 326 (76.5) | 209 (46.8) | 229 (62.1) | 219 (50.8) |
| Loss of smell/taste | 254 (15.2) | 110 (25.8) | 49 (11.0) | 42 (11.4) | 53 (12.3) |
| Cough | 505 (30.2) | 167 (39.2) | 107 (23.9) | 122 (33.1) | 109 (25.3) |

PEM = post-exertional malaise

*
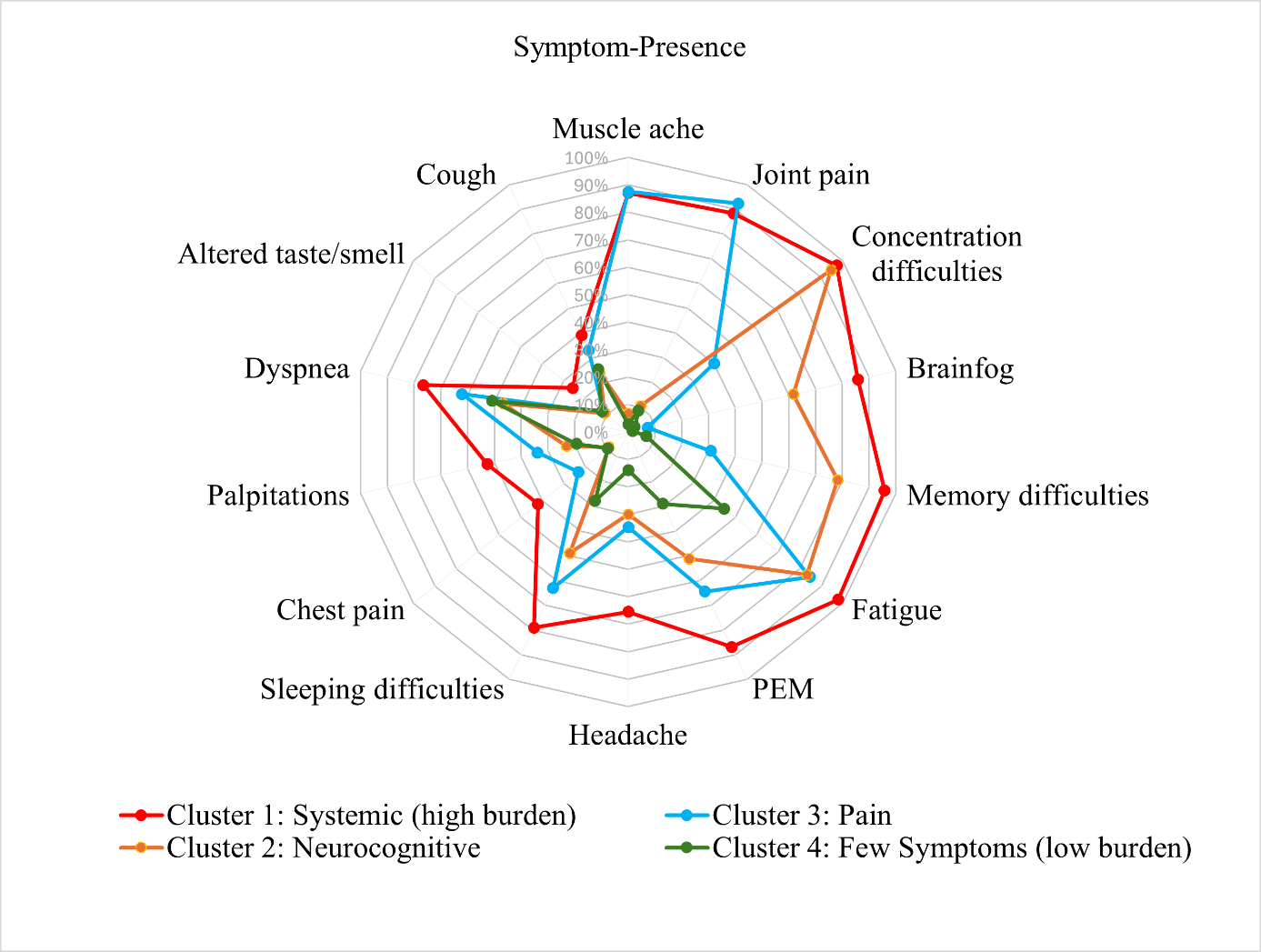
***Supplementary Figure 3.** Results of the two-step cluster analysis, including the *symptom-presence* variables: prevalence of the 14 symptoms across clusters. Cluster 1 – Systemic (high burden): N = 426; Cluster 2 – *Neurocognitive*: N = 447; Cluster 3 – *Pain*: N = 369; Cluster 4 – *Few Symptoms* (low burden): N = 431.

PEM = post-exertional malaise

**Supplementary Table 4.** FIT indices of the two-step cluster analysis, including the 14 *symptom-presence* variables (including only patients currently still affected).

| Number of Clusters | Schwarz’s Bayesian Criterion (BIC) | BIC Change ^a^ | Ratio of BIC Changes ^b^ | Ratio of Distance Measures ^c^ |
| --- | --- | --- | --- | --- |
| **1** | **29643.46** |  |  |  |
| **2** | **26280.84** | **-3362.61** | **1.00** | **2.03** |
| **3** | **24679.59** | **-1601.22** | **0.48** | **1.13** |
| **4** | **23277.44** | **-1402.14** | **0.42** | **2.09** |
| 5 | 22660.81 | -616.63 | 0.18 | 1.16 |
| 6 | 22144.01 | -516.80 | 0.15 | 1.27 |
| 7 | 21757.81 | -386.20 | 0.12 | 1.13 |
| 8 | 21427.32 | -330.50 | 0.10 | 1.27 |
| 9 | 21189.72 | -237.60 | 0.07 | 1.05 |
| 10 | 20968.86 | -220.86 | 0.07 | 1.04 |
| 11 | 20759.07 | -209.79 | 0.06 | 1.07 |
| 12 | 20569.21 | -189.86 | 0.06 | 1.03 |
| 13 | 20387.92 | -181.29 | 0.05 | 1.13 |
| 14 | 20238.21 | -149.71 | 0.05 | 1.04 |
| 15 | 20097.29 | -140.92 | 0.04 | 1.02 |

*Notes:* ^a^ The changes are from the previous number of clusters in the table. ^b^ The ratios of changes are relative to the change for the two-cluster solution. ^c^ The ratios of distance measures are based on the current number of clusters against the previous number of clusters. The cluster solution that best fits the data according to the fit indices is highlighted in bold.

**Supplementary Figure 4.** Results of the two-step cluster analysis, including the symptom-presence variables: graphical representation of predictor importance across the 14 symptom variables.


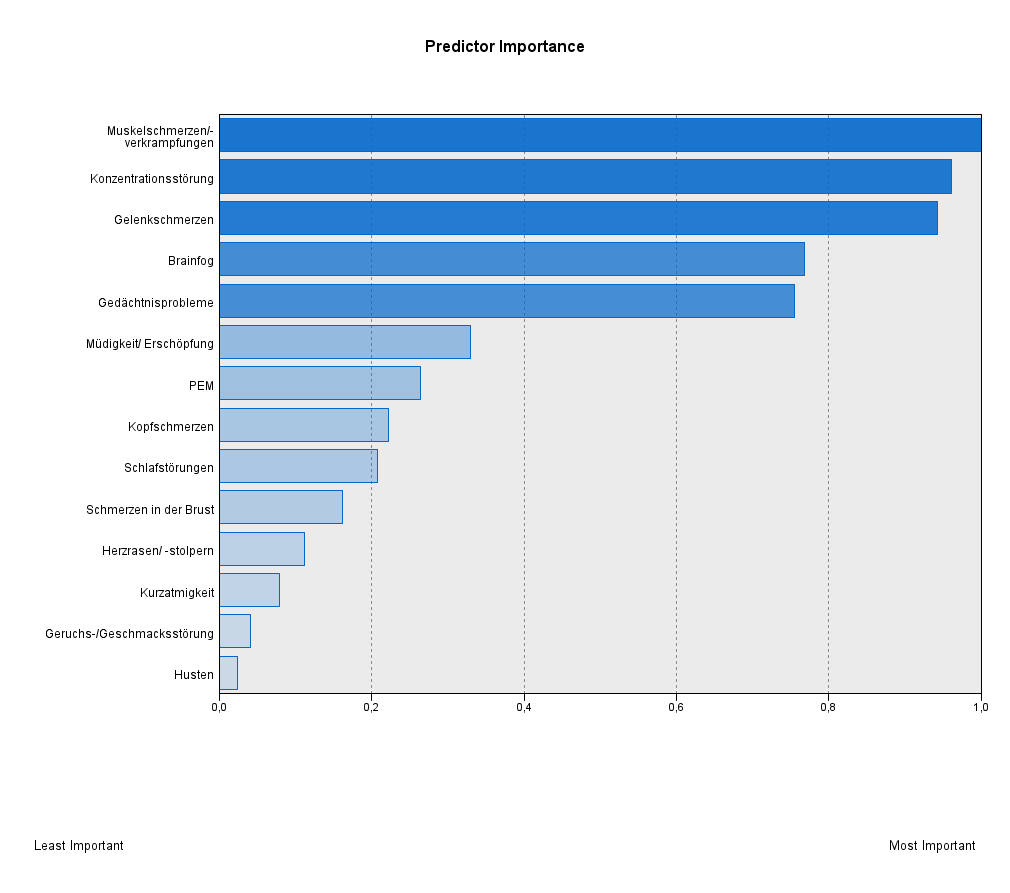


Muscle pain/cramps

Concentration difficulties

Joint pain

Brain fog

Memory difficulties

Fatigue/exhaustion

PEM

Headaches

Sleep disorders

Chest pain

Heart palpitations

Shortness of breath

Loss of smell/taste

Cough

**Supplementary Figure 5.** Results of the two-step cluster analysis, including the symptom-presence variables: graphical representation of cluster quality.

**
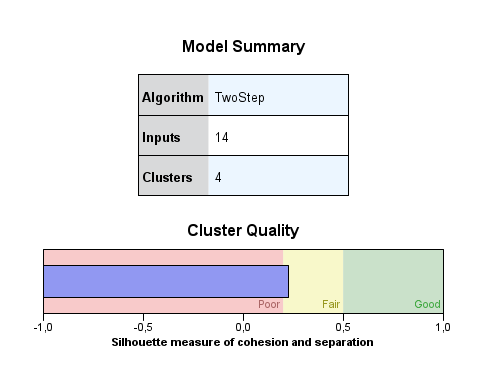
**

**Supplementary Table 5**. Comparison of the *symptom-presence* clusters regarding sociodemographic, clinical, and psychological continuous variables.

| Variable | Cluster | | | | Kruskal-Wallis-test (df=3  H, p, η² 95%-CI) |
| --- | --- | --- | --- | --- | --- |
| M (SD) | Systemic  (high burden)  N=426 | Neurocognitive  N=447 | Pain  N=369 | Few symptoms  (low burden)  N=431 |  |
| Age, years | 51.67 (11.24) | 48.97 (12.52) | 54.21 (11.40) | 50.71 (13.80) | H =30.87  p < .001  η²= 0.02  [0.01-0.03] |
| PHQ-4 | 6.83 (3.29) | 5.57 (3.07) | 4.66 (2.83) | 2.88 (2.52) | H =320.21  p < .001  η²= 0.20  [0.17-0.24] |
| PHQ-2 | 3.57 (1.75) | 2.88 (1.62) | 2.55 (1.55) | 1.50 (1.38) | H =310.94  p < .001  η²= 0.19  [0.16-0.23] |
| GAD-2 | 3.25 (1.78) | 2.69 (1.74) | 2.10 (1.55) | 1.37 (1.44) | H=256.30  p < .001  η²= 0.16  [0.12-0.19] |
| BS-6 | 17.07 (5.03) | 17.29 (5.21) | 17.44 (5.15) | 18.16 (5.59) | H=14.33,  p =.002  η²=0.01  [0.00-0.02] |
| KsT-5 | 11.56 (3.32) | 12.81 (3.33) | 12.92 (3.15) | 14.41 (3.01) | H=129.43  p < .001  η²= 0.09  [0.06-0.11] |
| CFS (total) | 10.33 (1.30) | 8.97 (2.26) | 7.88 (2.80) | 4.84 (3.21) | H=667.79  p < .001  η²= 0.40  [0.37-0.44] |
| CFS (physical) | 17.42 (2.84) | 14.60 (3.40) | 14.61 (3.53) | 10.71 (3.79) | H=557.49  p < .001  η² = 0.34  [0.31-0.38] |
| CFS (mental) | 9.27 (2.0) | 8.27 (2.19) | 6.18 (2.31) | 4.82 (2.12) | H=681.68  p < .001  η²=0.41  [0.38-0.45] |
| DSQ-PEM | 4.26 (1.29) | 3.01 (1.90) | 3.10 (1.86) | 1.29 (1.63) | H=481.39  p < .001  η²=0.29  [0.26-0.33] |
| Number of therapies | 3.32 (3.12) | 2.16 (2.52) | 2.25 (2.74) | 1.36 (2.13) | H=106.94  p < .001  η²=0.06  [0.04-0.10] |
| Number of specialists consulted | 3.52 (1.68) | 2.68 (1.51) | 2.71 (1.61) | 2.11 (1.26) | H=151.37  p < .001  η²= 0.09  [0.07-0.13] |
| Time since infection, months | 23.10 (8.80) | 22.48 (8.78) | 22.11 (8.37) | 20.64 (7.73) | H= 18.73  p < .001  η²= 0.01  [0.00-0.02] |
| Sick days due to infection | 223.27 (267.32) | 118.62 (209.06) | 112.82 (199.54) | 48.08 (97.75) | H= 138.49  p < .001  η²= 0.12  [0.09-0.16] |

*Notes.* PHQ-4: Patient Health Questionnaire-4; PHQ-2: Patient Health Questionnaire-2**;** GAD-2: Generalized Anxiety Disorder-2; BS-6: Brief Social Support Scale**.** KsT-5: Short Scale Measuring Perceived Social Participation; CFS: Chalder Fatigue-Scale; DSQ-PEM: DePaul Symptom Questionnaire – Post‑Exertional Malaise.

**Supplementary Table 6.** Comparison of the *symptom-presence* clusters regarding sociodemographic, clinical, and psychological categorical variables.

| Variable | Cluster | | | | Chi-square test (df=3  χ^2^, p, V, 95%-CI) |
| --- | --- | --- | --- | --- | --- |
| n (%) | Systemic  (high burden)  n=426 | Neurocognitive  n=447 | Pain  n=369 | Few symptoms  (low burden)  n=431 |  |
| Gender, f | 313 (73.5) | 331 (74.0) | 263 (71.1) | 291 (67.5) | χ^2^= 5.61  p = .132 |
| Unemployed | 132 (33.4) | 81 (19.5) | 96 (27.8) | 88 (22.6) | χ^2^ = 23.46  p < .001  V= 0.12  [0.07-0.17] |
| School, ≥ 12 years | 95 (24.7) | 126 (31.5) | 79 (24.1) | 115 (31.3) | χ^2^ = 9.05  p = .029  V= 0.08  [0.00-0.12] |
| Partnership, yes | 306 (79.1) | 315 (78.8) | 275 (83.8) | 305 (82.9) | χ^2^ = 4.82  p = .186 |
| Other chronic diseases, yes | 217 (56.1) | 180 (45.0) | 178 (54.1) | 150 (40.5) | χ^2^ = 24.31  p = < .001  V= 0.13  [0.07-0.17] |
| PHQ-4 ≥ 6 | 134 (49.4) | 87 (32.1) | 38 (14.0) | 12 (4.4) | χ^2^ = 216.25  p = < .001  V= 0.26  [0.22-0.29] |
| DSQ-PEM: Dead, heavy feeling after starting to exercise | 387 (90.8) | 301 (68.3) | 279 (77.3) | 156 (37.6) | χ^2^ = 310.0  p = < .001  V= 0.44  [0.39-0.48] |
| DSQ-PEM: Next day soreness or fatigue after non-strenuous, everyday activities | 345 (81.0) | 234 (53.2) | 230 (63.7) | 78 (18.8) | χ^2^ = 358.20  p = < .001  V= 0.47  [0.42-0.52] |
| DSQ-PEM: Mentally tired after the slightest effort | 360 (86.1) | 290 (65.8) | 165 (45.7) | 71 (17.1) | χ^2^ = 435.83  p = < .001  V= 0.52  [0.47-0.56] |
| DSQ-PEM: Minimum exercise makes you physically tired | 344 (82.3) | 252 (57.1) | 220 (60.9) | 119 (28.7) | χ^2^ = 247.52  p = < .001  V= 0.39  [0.34-0.44] |
| DSQ-PEM: Physically drained or sick after mild activity | 346 (82.8) | 251 (56.9) | 225 (62.3) | 112 (27.0) | χ^2^ = 270.18  p = < .001  V= 0.41  [0.36-0.45] |
| DSQ-PEM: Recovery time ≥ 14 hours | 159 (47.2) | 74 (22.0) | 67 (19.9) | 37 (11.0) | χ^2^ = 117.32  p = < .001  V= 0.27  [0.22-0.31] |
| CFS ≥ 4 | 418 (28.9) | 429 (29.6) | 334 (23.1) | 267 (18.4) | χ^2^ = 335.59  p = < .001  V= 0.45  [0.40-0.50] |
| Use of psychological therapy | 176 (43.7) | 139 (32.8) | 96 (27.2) | 68 (16.8) | χ^2^ = 72.08  p = < .001  V= 0.21  [0.16-0.26] |
| Use of active therapy | 150 (37.2) | 104 (24.5) | 83 (23.5) | 70 (17.3) | χ^2^ = 44.25  p = < .001  V= 0.17  [0.11-0.21] |
| Inpatient admission due to post-COVID | 49 (12.0) | 36 (8.3) | 37 (10.4) | 25 (6.1) | χ^2^ = 9.65  p = .022  V= 0.08  [0.01-0.12] |
| ICU due to infection | 24 (5.6) | 9 (2.0) | 11 (3.0) | 7 (1.6) | χ^2^ = 14.22  p = .003  V= 0.09  [0.03-0.13] |

*Notes:* CFS: Chalder-Fatigue-Scale; DSQ-PEM: DePaul Symptom Questionnaire – Post-Exertional Malaise. Psychological therapy includes relaxation therapy, psychological counseling, and psychotherapy. Active therapy includes rehabilitative sports and functional training/sports therapy.

**Supplementary Table 7.** Comparison of the *symptom-impairment* clusters regarding comorbid chronic conditions.

| **Chronic disease** | **Cluster** | | | | | **Chi-square test** (df=3  χ^2^, p) |
| --- | --- | --- | --- | --- | --- | --- |
| n (%) | Total  (n = 1673) | Systemic-  high burden  (n=461) | Neurocognitive  (n=489) | Pain  (n=419) | Few Symptoms-low burden  (n=304) |  |
| Asthma | 237 (15.9) | 85 (20.5) | 61 (16.3) | 54 (12.2) | 37 (14.5) | χ^2^ = 11.38  p = .010 |
| Chronic lung disease | 100 (6.7) | 35 (8.4) | 27 (7.2) | 25 (5.7) | 13 (5.1) | χ^2^ = 3.96  p = .270 |
| Heart disease | 63 (4.2) | 25 (6.0) | 19 (5.1) | 10 (2.3) | 9 (3.5) | χ^2^ = 8.48  p = .040 |
| Hypertension | 281 (18.9) | 91 (21.9) | 85 (22.7) | 71 (16.1) | 34 (13.3) | χ^2^ = 13.53  p = .004 |
| Stroke | 16 (1.1) | 5 (1.2) | 4 (1.1) | 5 (1.1) | 2 (0.8) | χ^2^ = 0.28  p = .964 |
| Chronic complaints in the lower back/lumbar spine | 238 (16.0) | 91 (21.9) | 72 (19.3) | 46 (10.4) | 29 (11.4) | χ^2^ = 28.12  p < .001 |
| Chronic complaints in the neck/cervical spine | 218 (14.7) | 90 (21.7) | 59 (15.8) | 55 (12.4) | 14 (5.5) | χ^2^ =35.60  p < .001 |
| Osteoarthritis | 189 (12.7) | 75 (18.1) | 56 (15.0) | 38 (8.6) | 20 (7.8) | χ^2^ =24.65  p < .001 |
| Diabetes | 88 (5.9) | 32 (7.7) | 19 (5.1) | 28 (6.3) | 9 (3.5) | χ^2^ =5.62  p = .132 |
| Chronic kidney disease | 24 (1.6) | 8 (1.9) | 7 (1.9) | 7 (1.6) | 2 (0.8) | χ^2^ =1.52  p = .678 |
| Elevated blood lipids or cholesterol levels | 92 (6.2) | 30 (7.2) | 21 (5.6) | 26 (5.9) | 15 (5.9) | χ^2^ =1.10  p = .778 |
| Depression | 167 (11.2) | 76 (18.3) | 28 (7.5) | 51 (11.5) | 12 (4.7) | χ^2^ =37.05  p < .001 |
| Anxiety disorder | 93 (6.3) | 42 (10.1) | 13 (3.5) | 28 (6.3) | 10 (3.9) | χ^2^ =17.87  p < .001 |
| Cancer | 41 (2.8) | 12 (2.9) | 10 (2.7) | 10 (2.3) | 9 (3.5) | χ^2^ =1.02  p = .799 |
| Epilepsy | 19 (0.7) | 1 (0.2) | 3 (0.8) | 4 (0.9) | 2 (0.8) | χ^2^ =1.66  p = .647 |
| Multiple sclerosis | 8 (0.5) |  | 2 (0.5) | 6 (1.4) | - | χ^2^ =9.17  p = .027 |
| Rheumatoid arthritis | 57 (3.8) | 25 (6.0) | 21 (5.6) | 7 (1.6) | 4 (1.6) | χ^2^ =18.23  p < .001 |
| Other | 285 (19.2) | 100 (24.1) | 63 (16.8) | 89 (20.1) | 33 (12.9) | χ^2^ =14.45  p = .002 |
